# Living with COVID-19: Subjective Well-Being in the Second Phase of the Pandemic

**DOI:** 10.1007/s10964-022-01648-8

**Published:** 2022-07-04

**Authors:** Golo Henseke, Francis Green, Ingrid Schoon

**Affiliations:** University College London, Institute of Education, LLAKES Centre, 20 Bedford Way, London, WC1H 0AL UK

**Keywords:** COVID-19, Stress Process Framework, Life Satisfaction, Panel Study, Adolescents and Emerging Adulthood

## Abstract

While there is ample evidence of the decline in mental health among youth during the onset of the COVID-19 pandemic, less is known about the determinants of recovery, which is the focus of this study. Drawing on a stress process framework, this study examines the associations of changes in direct, pandemic-related, and indirect, lockdown-related stressors with life satisfaction. A novel representative, longitudinal sample of British 16–25-year-olds is used, drawing on 6 data collections between February 2021 to May 2022 (N = 6000, 51% female, 24% ethnic minority, 46% in work, 35% with higher education). Using linear fixed-effects regression models, the findings suggest a substantial improvement in life satisfaction among youth. An increasing frequency of social contacts, receding worries about career prospects and job skills learning contributed significantly to increases in life satisfaction, whereas direct, health-related COVID-19 stressors did not affect life satisfaction. Sub-group analysis suggests that women’s, adolescents’, and students’ life satisfaction responded more strongly to the stressors considered in this study. The findings highlight the positive effects of less stringent lockdown restrictions, economic recovery, and opportunities for job skills learning on youth’s happiness.

## Introduction

The COVID-19 pandemic has had a profound impact on the lives of young people around the world. A rich research literature has documented a near-universal increase in clinically significant levels of depression, anxiety, and mental distress at the onset of the pandemic (Santomauro et al., 2021). However, the potential recovery in the wake of mass vaccination programmes when most COVID-19-related lockdown measures were lifted, and economic activity rebounded, has received less attention. This matters. First, previous studies suggested that levels of mental distress have remained elevated compared with pre-pandemic estimates but with notable heterogeneity across individuals (Fancourt et al., 2021). Second, there is no a priori reason to expect that determinants of recovery are the same as determinants of deterioration. Finally, life satisfaction, a crucial indicator of subjective well-being and healthy development in the transition to adulthood, has received less attention than symptom-based measures of mental distress in the rapidly growing literature around subjective well-being in times of COVID-19 (Pierce et al., 2021). Using a panel survey of 16–25-year-olds in Britain, this study examines (1) how young people’s life satisfaction has changed alongside symptom-based measures of mental health; (2) whether changes in pandemic-related stressors accounted for changes in life satisfaction; (3) if gender or the development stage in the transition to adulthood mattered for young people’s response to pandemic-related stressors.

### Life satisfaction as a dimension of mental health

Most scholars define mental health as a sense of subjective well-being and the capacity to effectively deal with and adapt to change and cope with environmental demands (Manwell et al., 2015). Within this framework, life satisfaction can be conceived as an important and distinct dimension of positive mental health and subjective well-being (Petersen et al., 2021). Life satisfaction refers to the cognitive evaluation of one’s life overall based on the fit between personal goals and achievement (Hall, 2014). Previous evidence supports a two-domain view of mental health where mental ill-health and subjective well-being are considered distinct constructs with only moderate overlap (Iasiello et al., 2020). Individuals can experience good well-being with some symptoms of mental ill-health (Lombardo et al., 2018). To understand improvements in youth’s subjective well-being in the aftermath of the pandemic, both dimensions, i.e., life satisfaction and mental ill-health, are considered in this study.

### The dynamics of life satisfaction and mental ill-health

There is general agreement that life events and changing circumstances can alter individuals’ short and long-term appraisal of their satisfaction with life as a whole (Diener et al., 2018) and their mental health (Thoits, 2010). It thus seems plausible that the pandemic-related restrictions on individual freedom, disruptions and uncertainties across multiple domains, including learning, careers, financial security, social interactions, one’s health and the health of family and friends, have shaped young people’s subjective well-being. Previous studies have documented significant differences in life satisfaction trends due to varying experiences of pandemic and lockdown-related disruptions (Preetz et al., 2021).

Lockdown measures have become less stringent since the start of 2021 across OECD countries and regions (Hale et al., 2021). The UK government lifted most restrictions for England on 19 July 2021. The Scottish and Welsh governments did the same by 8 and 9 August, respectively, and the Northern Ireland executive lifted most restrictions on 16 August. The last restrictions in England were phased out on 26 February 2022. Fewer restrictions meant that people regained control over their daily activities, including education, work, leisure, and socialising with peers. Effective vaccines reduce health risks from COVID-19. The economic recovery helped reduce employment insecurity (Dias et al., 2021). However, health and safety concerns and the spectre of new variants contribute to continued uncertainties regarding health and the delivery of learning (Raybould, 2021), employment, and opportunities for learning job skills in work or education (Green et al., 2021a).

Besides the current study, one other study also examined trends in young people’s life satisfaction after the launch of mass vaccine programmes in 2021 using US panel data (Graupensperger et al., 2022). The study documents significant associations of pandemic-related stressors, particularly experiences of strain on social interactions, with within-person trends in well-being up until August 2021. However, it remains unclear how well their results generalise to other contexts and over time.

### Sources of Stress

Guided by the stress process framework within a life-course approach (Pearlin et al., 2005), this study distinguishes between discrete life events and chronic stressors. Stress is understood not as a monolithic concept but rather as an ongoing process that involves interactions between a changing individual and a changing context. Chronic stressors reflect experiences of prolonged hardship or discrimination, for example, associated with socio-economic status. Discrete life events generally refer to normative events, such as the assumption of new social roles in the transition to adulthood and unexpected or non-normative life events, such as the disruptions caused by the COVID-19 pandemic. The pandemic, in turn, can influence individual lives directly (e.g., illness) or indirectly through lockdown restrictions. Life events hold the potential to amplify pre-existing or dormant stressors and introduce new stressors, a process described as stress proliferation (Pearlin et al., 2005). The notion of stress proliferation implies that mental health disparities result from various factors, some of which are already in place before the onset of distinct stressors. This study focuses on the onset or end of discrete, potentially stressful life events that occurred within the family or concerned the self. Stressors are conceptualised as common and salient adverse experiences or worries due to the pandemic or related behavioural restrictions that can disrupt the fit between individual and environmental characteristics (Núñez-Regueiro and Núñez-Regueiro, 2021). This study focuses on direct pandemic-related health effects and indirect lockdown-related effects, including career-related stressors, social stressors referring to the frequency of social contact, and financial stressors (Graupensperger et al., 2022).

Direct COVID-19-related stressors include a positive diagnosis of being infected with the virus and the experience of severe illness or the death of close family members or friends. Although young people are generally at a lower risk of COVID-related morbidity and mortality (Banerjee et al., 2020), the vaccine programme shifted the disease burden to unvaccinated, usually young people, during the gradual rollout (Mallapaty, 2021). Diagnosis with COVID-19 has been found to predict mental distress (Taquet et al., 2021), but the association attenuated with time (Chandola et al., 2020).

Salient, indirect, lockdown-related stressors arise from uncertainties regarding learning, career prospects or financial circumstances, and limitations on social interactions (Robinson and Daly, 2021). For example, school closures, changes to examinations, or loss of employment are linked to reduced social contacts and worries about future employment, career prospects and financial security. While the pandemic and associated uncertainties disrupted career planning for many workers, young adults with often little experience in handling adversities might have struggled more than older adults to adjust to the constraints on their agency and outlook on the future (Settersten et al., 2020). Lack of social contacts and loss of control with whom to socialise, either voluntary as a response to COVID-19 or enforced by policies, are thought to be major determinants for the decline of mental health during the early stages of the pandemic (Loades et al., 2020). Financial hardship is an undesirable state that can limit individual agency, self-esteem, and feeling of coping and, in doing so, reduce mental health (Wright et al., 2020). With job vacancies being in free fall during 2020, the pandemic severely interrupted young people’s opportunities to gain experience in the workplace (Holt-White and Montacute, 2020), with potentially adverse consequences for identity, ambitions, future worries, and thus mental health (Fouad and Bynner, 2008). Recessions affect young people who are on the cusp of establishing themselves in the job market (Schoon and Bynner, 2019). Uncertain transitions and stunted job skills learning can permanently scar career development (Liu et al., 2016), something young people are well aware of (Green et al., 2021a).

Stressors can alter mental health –both ill-health and well-being – by changing experiences of the present, for example, by increasing the time spent in undesirable states, altering future expectations, or leading to a re-evaluation of past experiences (Durayappah, 2010). But despite the close association across mental health domains, mental distress and well-being can react differently to specific stressors (Patalay and Fitzsimons, 2018). Going beyond previous research on individuals’ mental health during the COVID-19 lockdown, this study uses longitudinal data from a population-representative sample of young people to assess within-person changes in life satisfaction and their relationship with changes in direct and indirect stressors and mental distress. Understanding the contribution of pandemic-related stressors on life satisfaction is essential to assess the potential longer-term effects on young people’s subjective well-being. Poor life satisfaction can reduce functioning in the short term and impair future well-being and economic outcomes in the long term (Sellers et al., 2019).

### Confounding influences

As discussed above, life satisfaction will change not only with pandemic-related stressors but also with important, normative life events over the life course. For example, life satisfaction changes with age during the transition to adulthood (Henkens et al., 2022). More generally, there can be considerable heterogeneity in experiences and stress response, as the notion of youth spans a time frame of 10 years between ages 16 to 25, which covers a wide range of developmental progressions, including the transition from school to work and to independent living. Employment benefits life satisfaction as young people move from education into their careers (Gagné et al., 2022). During most of the pandemic, the UK’s job retention schemes provided income support and reduced job insecurity for incumbents. At the same time, young people in education faced lost learning from school closures, an uneven move to online learning, disruptions to exams and an uncertain outlook after graduation (Green et al., 2021a). Leaving the parental home is another central marker of adulthood (Sharon, 2016). Economic uncertainty can delay and reverse residential independence with potentially adverse consequences for life satisfaction and mental health (Evandrou et al., 2021). Furthermore, social support is a resource that can boost well-being directly as well as through other psychosocial resources factors such as self-efficacy and optimism, well-being and psychosocial adjustment (Schoon and Henseke, 2022). Assessing the consequences of stressors on life satisfaction will require accounting for these potentially confounding influences.

Moreover, while this study conceives stressors as common and salient adverse experiences or worries, individuals might weigh them differently in their appraisal of life satisfaction. The extant literature documents relevant differences in the formation of life satisfaction by gender and developmental phase, including adolescence versus emerging adulthood, completing education and entering work, or leaving the parental home (Gagné et al., 2022). There are inconsistent findings regarding gender differences in life satisfaction. Pre-pandemic, women tended to report greater overall life satisfaction than men across countries (Blanchflower and Clark, 2021). Still, these gender differences appear to have been changing during the pandemic (Blanchflower and Bryson, 2022). Those aged 18 or older tend to be less satisfied than younger people (Henkens et al., 2022), those in education report higher levels of life satisfaction than those not in education (Burger and Samuel, 2017), and young adults living independently are more satisfied than those living with their parents (Gagné et al., 2022). To what extent these factors shape the relative weight of stressors for life satisfaction in adolescence and emerging adulthood during the pandemic has received limited attention. There is, however, some general evidence suggesting that economic insecurity is more threatening for men than women (Kopasker et al., 2018) and that girls are more sensitive than boys to interpersonal stressors related to interactions with friends and family (Henkens et al., 2022).

## Current Study

Drawing on the previous literature, this study tests if life satisfaction has improved and if the prevalence of pandemic-related stressors has declined since February 2021 (Hypothesis 1). Next, the relationship of changes in stressors with changes in life satisfaction is examined within individuals. According to the stress process framework, it is expected that the considered stressors have a combined effect on life satisfaction conditional on normative life events over the life course approximated by the covariates (Hypothesis 2a). Moreover, based on previous research, it is anticipated that particularly social relationship stressors will have a salient impact on the well-being of young people (Hypothesis 2b). To assess the magnitude of different stress effects on life satisfaction, their effect is compared to the impact of an increase in mental distress on life satisfaction. Finally, assuming heterogeneity in stress responses, it is expected that the impact of stressors on life satisfaction will vary across developmental stages (age, education, living independently) and gender (Hypothesis 3). Accordingly, the study will test if the stressor coefficients are stable across socio-demographic groups or if there is a stress proliferation in distinct subgroups.

## Methods

### Data

The study uses longitudinal, individual-level data from the six waves of the Youth Economic Health Monitor (YEAH) survey, collected quarterly between February 2021 and May 2022. The YEAH survey was a quota panel study of 16-25-year-old UK residents with 1,000 observations per wave recruited from web access panels managed by Ipsos Mori and partners. It collected quarterly information on life satisfaction and mental health alongside data on education, work, career readiness, skills development, and future expectations. For the initial sample, quotas were set according to age by gender, working status and region. The sampling approach was chosen to recruit a balanced sample of a usually difficult-to-reach demographic during the second wave of the pandemic. Moreover, given the lockdown restrictions at the time, CAWI was the only feasible data collection mode.

In total, the sample contains 6,000 cases from 3,746 individuals. Invited panellists received survey information (including survey duration and incentive points), a unique URL to access the questionnaire, a physical address for Ipsos Mori, a member support email address, a link to the privacy policy, and opt-out information. Ipsos used a mix of points and sweepstakes to incentivise survey participation.

Table A1 in the appendix compares descriptive statistics of the sample against sample statistics from the UK Household Longitudinal Study (UK-HLS) COVID-19 surveys: a pivotal source to track population well-being in the UK (e.g., Pierce et al., 2020), and population estimates from the Quarterly Labour Force Survey 2021 (LFS), where data availability permits. The LFS is the largest continuous random probability survey in the UK. In all, compared with the UK-HLS COVID-19 survey, the YEAH sample was less female, subjectively less well-off, and less likely to live with their parents. YEAH, and UK-HLS COVID-19 were slightly less white and had a higher prevalence of self-reported limiting health conditions than the LFS population estimates suggested. Overall, the sample is relatively close to the estimated demographic composition from the LFS, albeit with some deviations, for example, by educational attainment or living arrangement. The quota approach thus yielded a partially balanced sample.

### Measures

#### Life satisfaction

Life satisfaction, the dependent variable, was measured using a single item: “Overall, how satisfied are you with your life nowadays?” with response options ranging from 0 “Not at all satisfied” to 10 “Completely satisfied”. The question is part of the UK Office for National Statistics’ well-being measures, underwent careful cognitive testing, and is used in numerous social surveys (ONS, 2018). The single-item assessment performs similarly to multi-item life satisfaction measures across various contexts and has been validated repeatedly in samples of young people (Levin and Currie, 2014).

#### Mental distress

Symptoms of mental distress were assessed using a short-form Hopkins Symptom Checklist (HSCL-5), a five-item self-reported scale designed to yield a brief evaluation of worry, anxiety and dysphoria in general population surveys (Strand et al., 2009). HSCL-5 has shown good reliability as a measure of depression and anxiety with satisfactory construct validity (Schmalbach et al., 2021). The instrument asks respondents to report how much they were bothered by feelings of fearfulness, nervousness, hopelessness, sadness and worries in the week before the survey, with responses ranging from 1 “Not at all” to 4 “Extremely”. The row means over the five items form an index of mental distress (ω = 0.88).

#### Stressors

##### Direct, pandemic-related stressors

Direct stressors were assessed with a question about a *positive diagnosis* of the illness and the *experience of severe illness* *or the death* of close family members or friends: Which, if any, of the following have occurred as a direct or indirect result of the coronavirus pandemic? (1) Serious illness of a close family member or friend; (2) death of a close family member or friend; (3) I have been tested for coronavirus (COVID-19) and received a positive diagnosis; (4) none of these. If the respondent answered positive to any of the options 1 to 3, the presence of a direct stressor was noted.

##### Indirect, lockdown-related stressors

Measures of indirect stressors included the assessment of *reduced social contacts*, *perceived financial strain*, *concerns about financial future*, *career prospects*, and *job skills learning*. To measure the frequency of social contacts, respondents were asked: “Has the amount of social interaction you have with friends, relatives or colleagues who you don’t live with changed since the start of the FIRST lockdown on 23^rd^ March 2020?” Response options ranged from (1) “A lot more than in March 2020” to (5) “A lot less than in March 2020”. An indicator variable was defined when the respondent reported a lot fewer social interactions. Perceived financial strain was assessed with a question about their current financial situation: “All things considered; how well would you say you yourself are managing financially these days?” with responses ranging from (1) “Living comfortably” to (5) “Finding it very difficult”. A binary indicator was coded to take the value of one if survey participants were finding their financial situation difficult. In addition, respondents were also asked: “Looking ahead, how do you think you yourself will be financially a year from now, will you be…” with responses ranging from (1) Better off; (2) Worse off than now; (3) About the same. A binary variable was defined as equal to one if respondents expected their financial situation to worsen in the future. Career concerns were assessed with two items asking participants to evaluate how the coronavirus pandemic affected their career prospects and opportunities for job skills learning. Respondents answered on a five-point scale from 1 “Worsened a lot” to 5 “Improved a lot”, with “don’t know” as an additional response option. Binary indicators were defined if respondents felt the pandemic worsened their career prospects and opportunities for job skills learning a lot, respectively. While not an objective account of skill loss, the variables have shown meaningful correlations with predictors of learning (Green et al., 2021b).

#### Covariates

##### Time trend

To measure trends, a set of survey wave dummy variables was used. The reference was February 2021, when the first survey wave was collected.

##### Time-varying covariates

To account for confounding influences from potentially significant changes and interruptions in young people’s transition to adulthood, the analysis controls for *age*, *employment status, living arrangements*, and *social support*. Age and age squared control for the non-linear relationship with life satisfaction. To account for changes in employment status, the empirical models include an indicator if respondents were in paid work. Finally, the number of people with whom respondents can discuss intimate and personal matters was included to account for changes in social support.

##### Time-invariant covariates

Stressor variables were interacted with a set of time-invariant control variables to assess the homogeneity of the life satisfaction response to the here considered stressors. More specifically, sub-group analyses were carried out to test if the associations of stressors with life satisfaction were invariant by *gender* (male/ female), *age group* (16–18, 19+), *employment at baseline* (yes, no), and *living with parents* (yes, no).

### Analytical strategy

To answer the research questions, this study estimates linear fixed-effects models. Fixed-effects models use the variation within individuals over time to estimate parameters. In so doing, fixed-effects models control for time-invariant influences even if these are unobserved or correlated with the experience of or worries about stressors. By comparing changes within the same individual over time, a fixed-effects model removes potentially confounding time-invariant factors such as exposure to chronic stressors, stress resilience, personality, coping resources, history of mental health, or genetics (Cameron and Trivedi, 2010).

Moreover, fixed-effects models remove potential method bias if the underlying factors are time constant. Since the survey waves were about three months apart, this assumption may not be too far-fetched. Because the fixed effects will absorb all other time-invariant factors, including family background or chronic stress exposure, it is impossible to identify these factors’ role in life satisfaction. However, as described above, tests were conducted to assess if stress pathways differed across subgroups by introducing interaction terms between stressors with time-invariant covariates in the estimation models. Wald tests of the joint significance of the interaction terms assess if the correlation between life satisfaction and the measured stressors differed across subgroups. Fixed-effects models have proven helpful in illuminating the responsiveness of well-being to stressors during the pandemic (e.g., Chandola et al., 2020).

A baseline fixed-effects model with the time-variant control variables and period dummies is estimated to meet the first objective. The stressor variables are added to the baseline model to answer the second objective. The coefficients measure the score change in life satisfaction associated with the experience or perceptions of stressors conditional on the other variables in the model. Beyond single coefficients, a series of Wald tests were used to assess whether direct and indirect stressors were jointly associated with life satisfaction. The measure of mental distress was added to the specification in a third estimation model to provide a benchmark for the estimated stressor effects. Compared with the baseline model, changes in the coefficients of the survey period dummies indicate how much of the time trend in life satisfaction is attributable to the stressor variables. For the third objective, a set of interaction terms of stressors with the time-invariant controls (one at a time) was included in the fixed-effects model, testing if these interaction terms are jointly statistically significant. Rejection of the null hypothesis is taken as evidence for heterogeneous stress responses.

Like other longitudinal studies of young people, the panel sample displays noticeable levels of attrition: respondents fail to participate in one or more waves and item nonresponse for variables within each wave (Jeličic et al., 2010).

To tackle item nonresponse, multiple imputation was used by implementing chained equations (MICE) for all variables in this study in 30 samples in Stata 17 (Royston and White, 2011). By imputing missing values, the study uses 100 % of the sample.

To deal with panel attrition, we rely on the properties of the fixed-effects models. The proposed fixed-effects approach ‘controls’ for observed or unobserved determinants of attrition if survey participation is due to time-invariant factors (Jones et al., 2013). It is, however, conceivable that participants leave the panel due to time-varying factors such as a spell of poor (mental) health, acute stress, or other significant life events. In robustness checks, a test for attrition bias was carried out by adding an indicator for survey participation at *t* + 1 to the estimation model at *t* (Jones et al., 2013). A non-significant coefficient is evidence against attrition bias.

## Results

### Descriptive Trends

Life satisfaction varied substantially through the pandemic, with low points during periods of lockdown restrictions.

**Fig. 1:**
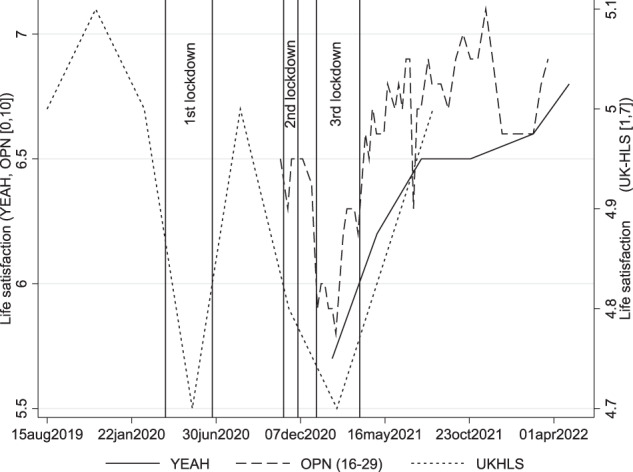
Life satisfaction before and during the COVID-19 pandemic in the UK --- A multi-survey comparison. Mean life satisfaction estimates from the full YEAH sample, published statistics from the ONS Opinions and Lifestyle Survey (COVID-19 module) for 16-29-year-olds, and the UK-HLS COVID-19 and mainstage surveys. UK-HLS are projected on the right vertical axis

Figure 1 compares trends in life satisfaction from the YEAH survey (solid line) with published statistics from the Opinions and Lifestyle Survey (OPN, dashed line) and secondary data from the UK-HLS COVID-19 study (dotted line). OPN was a high-frequency, repeated cross-sectional study undertaken by the Office for National Statistics to understand the impact of the COVID-19 on British society (ONS, 2022). The statistics refer to 16-29-year-olds. Unlike the other sources, UK-HLS permits a comparison with pre-pandemic life satisfaction. It measures overall life satisfaction on a 7-point scale. The mean values are thus projected on the secondary vertical axis in Fig. 1.

According to the YEAH survey, there has been a clear improvement in mean life satisfaction by a scale point between February 2021, the middle of the 3^rd^ national lockdown in the UK, and May 2022. The trend towards better life satisfaction in the second phase of the pandemic after the third lockdown was consistent across surveys with minor differences. YEAH and OPN agreed on start and end values, but average life satisfaction figures in YEAH improved less sharply than in OPN. OPN estimates peaked at 7.1 at the end of November 2021 before falling back and plateauing between 6.6 and 6.9. According to UK-HLS estimates, young people’s life satisfaction had improved to pre-pandemic levels in the UK by the autumn of 2021.

### Hypothesis 1 Trends in Life Satisfaction, Mental Distress, and Stressors

Table 1 reports changes in life satisfaction, mental distress and pandemic-related stressors. As noted above, life satisfaction improved over the six survey waves. The mean difference between February 2021 and May 2022 was highly statistically significant. By contrast, mental distress scores did not improve systematically: we measured the same mean HSCL-5 score at the start and the end of the survey period.

**Table 1:** Trends in life satisfaction, mental distress, direct and indirect stressors from February to October 2021 in a sample of 16–25-year-olds in the UK

	Feb-21	May-21	Jul-21	Oct-21	Feb-22	May-22	Δ
**Well-being**							
Life satisfaction	5.72	6.24	6.46	6.55	6.55	6.75	1.04^***^ (0.096)
Mental distress	2.32	2.27	2.26	2.23	2.25	2.32	−0.00 (0.036)
**Direct stressors**							
COVID-19 diagnosis	0.09	0.10	0.13	0.17	0.34	0.40	0.31^***^ (0.019)
COVID-19 among family/friends	0.30	0.26	0.25	0.28	0.29	0.29	−0.01 (0.021)
**Indirect stressors**							
Reduced social contact	0.55	0.39	0.32	0.22	0.21	0.18	−0.37^***^ (0.020)
Financial strain	0.17	0.16	0.15	0.14	0.17	0.17	0.01 (0.017)
Financial worries	0.17	0.17	0.15	0.16	0.19	0.22	0.05^**^ (0.018)
Career worries	0.21	0.16	0.13	0.08	0.10	0.08	−0.13^***^ (0.016)
Job skills worries	0.17	0.14	0.14	0.10	0.11	0.10	−0.07^***^ (0.015)

N = 6000 sample of 16-25-year-olds. Weighted mean values using the provided survey weights. Missing values imputed using 30 multiple imputations. Autocorrelation and heteroskedasticity robust standard errors in parentheses

^*^p < 0.05, ^**^p < 0.01, ^***^p < 0.001

Stressors have taken different trajectories as well. On the one hand, the percentage of young people who had received a positive COVID-19 diagnosis more than quadrupled from nine per cent in February 2021 to 40 per cent by May 2022. Most of this increase occurred after October 2021. However, the experience of severe COVID-19 cases among family and friends stabilised at about 30 per cent. COVID-19 remained prevalent.

On the other hand, some indirect stressors declined substantially, consistent with less stringent behavioural restrictions and a recovering economy. In February 2021, more than half of the respondents reported meeting with others less frequently than before March 2020. This fraction had dropped to less than one in five by May 2022. Similarly, in May 2022, eight per cent worried about the pandemic’s impact on their career prospects compared to 21 per cent fifteen months earlier. Likewise, there was a drop in the proportion of young people who worried about the pandemic’s impact on job skills learning from 17 per cent in February 2021 to ten per cent in May 2022.

However, subjective financial strain was about as common in May 2022 as in February 2021. Worries about the individual financial situation in a year’s time had become more frequent by the end of the observation period.

In all, the statistics give a mixed picture. As expected, life satisfaction improved, and there was an increase in social contacts and a reduction of career-related stressors, including fewer concerns about career prospects or job skills training. However, levels of mental distress did not change, the direct impact of the pandemic on health did not decrease, financial strain remained constant and financial worries rose. Hypothesis one receives thus only partial support.

### Hypothesis 2: The Association of Stressors with Life Satisfaction

Table 2 summarises the main findings from a fixed-effects regression of life satisfaction on survey period dummies, stressors, and time-varying covariates.

**Table 2:** Fixed-effects regression of stressors on life satisfaction (N = 6000)

	(1)	(2)	(3)
**Survey period (Ref: Feb-2021)**			
May 2021	0.504^***^	0.452^***^	0.438^***^
	(0.087)	(0.087)	(0.087)
August 2021	0.517^***^	0.439^***^	0.416^***^
	(0.097)	(0.099)	(0.098)
October 2021	0.604^***^	0.479^***^	0.426^***^
	(0.105)	(0.109)	(0.108)
February 2022	0.530^***^	0.435^***^	0.375^**^
	(0.121)	(0.127)	(0.125)
May 2022	0.471^***^	0.358^*^	0.323^*^
	(0.140)	(0.149)	(0.146)
**Mental distress**			
HSCL5 score			−0.535^***^
			(0.073)
**Direct Stressors**			
COVID-19 diagnosis		−0.174	−0.174
		(0.108)	(0.109)
COVID-19 among family/friends		−0.142	−0.121
		(0.092)	(0.090)
**Indirect Stressors**			
Reduced social contact		−0.221^**^	−0.225^**^
		(0.078)	(0.077)
Financial strain		−0.083	−0.011
		(0.110)	(0.107)
Financial future worries		−0.066	−0.064
		(0.087)	(0.084)
Career worries		−0.280^*^	−0.231
		(0.121)	(0.118)
Job skills worries		−0.364^**^	−0.330^**^
		(0.128)	(0.127)
F-test (all stressors)		4.55	4.08
*p* value		0.000	0.000
F-test (direct stressors)		2.25	1.92
*p* value		0.106	0.147
F-test (indirect stressors)		5.66	5.15
*p* value		0.000	0.000

Estimates from linear fixed-effects regression models of life satisfaction on period dummies, stressor variables and time-variant controls in a multiple imputed sample of 16–25-year-olds UK residents (Observations = 6000; Groups = 3746). Control variables include age in years (linear, squared), living with parents, student status, and social support. Autocorrelation robust standard errors in parentheses

^*^p < 0.05, ^**^p < 0.01, ^***^p < 0.001

Column 1 reports a baseline model with period dummies conditional on the covariates and individual fixed effects. The estimates confirm that life satisfaction improved markedly in the study period. The mean score in October was 0.6 points above its score in February 2021. Unlike the descriptive trends, the estimates suggest a drop in life satisfaction between October 2021 and May 2022. Nonetheless, compared with the baseline in February 2021, mean life satisfaction had improved by nearly 0.5 points within individuals.

Column 2 presents the headline estimates. All stressors entered the specification with the expected sign and jointly explained a significant fraction of the variation in life satisfaction within individuals (F(7,3734)=4.55, p = 0.000). Life satisfaction was negatively associated with reduced social contacts (b = −0.221, p = 0.004), worries about career prospects (b = −0.280, p = 0.020), and worries about job skills learning (b = −0.364, p = 0.005). By contrast, there was no firm evidence that direct pandemic-related stressors either individually or jointly predicted changes in life satisfaction (F(2,3667.7) = 2.25, p = 0.106). In all, receding experience and perceptions of the here considered stressors explained about 24 per cent (((0.471–0.358)/0.471) × 100%) of the within-individual difference in life satisfaction between February 2021 to May 2022; thus supporting hypothesis 2a.

Adding mental distress to the specification attenuated some of the estimated effects without changing the substantial results (column 3). A point increase in the mental distress score was associated with a highly significant 0.54-point decline (p = 0.000) in life satisfaction. The individual stressors that emerged as significant predictors in column 2 remained statistically significant below or very close to common levels. The coefficient of mental distress helps benchmark stressors’ influence on life satisfaction. According to the model estimates, the effect of reduced social contact on life satisfaction was equal to 42 per cent of a unit increase in the mental distress index (−0.224/−0.535, p = 0.005), for career worries 43 per cent (p = 0.064) and job skills concerns about 62 per cent (p = 0.015), respectively. These are substantive effects. In combination, changes in the considered stressors and mental distress accounted for 32 % of the improvement in life satisfaction within individuals between February 2021 and May 2022.

The estimation results support H2a and H2b. Close to a quarter of the improvements in life satisfaction between February 2021 and May 2022 could be attributed to receding stressors. Social interactions emerged as a salient stressor for life satisfaction together with career worries (worries about prospects and job skills learning). At the same time, there was no firm evidence that the direct pandemic-related stressors influenced life satisfaction systematically in this period.

### Hypothesis 3: Heterogeneous Stress Responses by Gender and Developmental Stage

As discussed earlier, effect heterogeneity was tested by gender (female, not female), age (16–18, 19+), education status (in education, not in education), and living with parents (yes, no) – all measured when survey respondents first joined the panel.

**Fig. 2:**
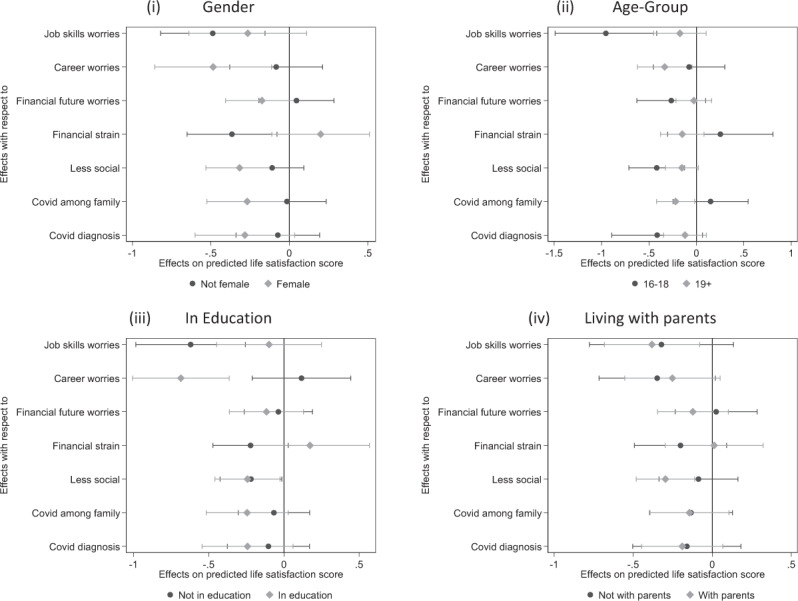
Assessing subgroup differences in the association of stressors with life satisfaction. (i) Gender (ii) Age-Group (iii) In Education (iv) Living with parents

Table 3 reports the results from the Wald tests. Recall that these tests assessed whether the interaction terms of stressor variables with sub-group indicators were jointly significant in the fixed-effects specification without mental distress. Rejection of the null hypothesis is evidence for heterogeneous stress responses across these groups. Figure 2 displays the estimated coefficients for each stressor by subgroup and their confidence intervals.

**Table 3:** Subgroup differences in the association of stressors with life satisfaction

Subgroup	Life Satisfaction
Female (no, yes)	F(7, 3729.8) = 2.32 p value = 0.023
Age (16-18, 19+)	F(7, 3735.8) = 2.18 p-value = 0.033
Studying (no, yes)	F(7, 3731.7) = 2.65 p-value = 0.010
Living with parents (no, yes)	F(7, 3735.1) = 0.46 p-value = 0.864

Test statistics and p-values from composite Wald tests of the interaction terms between stressors and subgroup indicators in linear fixed-effects regression models. The dependent variable is life satisfaction. Explanatory variables include period dummies, stressor variables, their interaction terms with subgroup indicators, and time-variant controls. Estimates from a sample of 16–25-year-olds UK resident with multiple imputed missing values

According to the estimates, the effect of stressors varied overall by gender, age group and education status, but not by living arrangement. The following summarises statistically significant sub-group differences. Young women’s life satisfaction responded more strongly to the experience of COVID-19 among family or friends (b = −0.268, p = 0.04 vs b = −0.015, p = 0.906) and to career worries (b = −0.486, p = 0.010 vs b = −0.084, p = 0.580), but less to financial strain (b = 0.2, p = 0.208 vs. −0.366, p = 0.012) than those life satisfaction who did not identify as female. For adolescents aged 18 years or less, worries about their job skills learning (b = −0.955, p < 0.001 vs b = −0.174, p = 0.213) weighed more heavily in their evaluation of life satisfaction than among their older peers. For youth in education at baseline, life satisfaction reacted especially to career worries (b = −0.685, p < 0.001 vs b = 0.117, p = 0.458), whereas worries about job skills learning emerged as greater adversity among those not in education (b = −0.62, p = 0.001 vs b = −0.01, p = 0.578). In all, the results point toward relevant differences in the relationship between COVID-19-related stressors and life satisfaction across life stages supporting H3.

### Sensitivity Analysis: Testing for attrition bias

This final analytical section tested for attrition bias. As detailed above, a test for potential bias due to panel attrition was carried out by adding an indicator variable for participation in the subsequent survey wave to the preferred model specification. The coefficient of the added indicator was small and statistically insignificant (b = −0.142, p = 0.592). There is thus no strong evidence for attrition bias in the fixed-effects models. The main findings should be robust to panel attrition.

## Discussion

Little is known about the potential improvements in youth’s subjective well-being, including life satisfaction, in the second phase of the pandemic when vaccine programmes were rolled out, and behavioural restrictions were lifted. By assessing the relationship between changes in pandemic-related stressors with changes in life satisfaction among adolescents and emerging adults, this study aimed to illuminate the process of recovery. Drawing on longitudinal data from a survey of young people from February 2021 to May 2022, three central findings emerged. Firstly, life satisfaction had improved substantially since its temporal low-point in February 2021; Secondly, more frequent social contacts and declining worries about career prospects and job skills learning contributed significantly to greater life satisfaction, whereas direct, health-related COVID-19 stressors did not. In total, nearly a quarter of the difference in life satisfaction between February 2021 to May 2022 was accounted for by receding stressors. Thirdly, the influence of stressors on life satisfaction was not homogenous among youth but varied by gender and aspects of their developmental stage (age-group, education status).

Secondary data from other UK-wide surveys corroborate the trend toward greater life satisfaction among adolescents and young adults (H1). Estimates using combined UK-HLS data with data from the UK-HLS COVID-19 studies suggested that life satisfaction had recovered to pre-pandemic levels by the autumn of 2021. By contrast, there are no apparent changes in self-reported symptoms of depression/ anxiety. While life satisfaction and mental distress were not independent, the contradicting trends demonstrate the value of a multi-dimensional conceptualisation of subjective well-being.

Trend patterns for the stressor were varied. The percentage of young people who were ever diagnosed with COVID-19 quadrupled, while the experience of severe cases of COVID-19 among friends and family remained stable. Following the reduction of lockdown restrictions in July 2021, there was a significant increase in social contacts and reduced concerns about career prospects and job skill learning until October 2021, with no notable change after that. Finance-related stressors remained stable throughout most of the observation window but rose in the final months, which coincided with a period of unusually high inflation and a cost-of-living crisis.

The fixed-effects results document that, as expected (H2), individual life satisfaction responded to changes in stressors. Disruptions to social interactions and the receding career-related stressors, including worries about career prospects and lost job skills learning, were significant predictors of life satisfaction in this recovery period. According to the current study direct, illness-related stressors were neither individually nor jointly associated with within-person changes in life satisfaction, which might explain why life satisfaction did not worsen over the winter of 2021-22 despite the surge in infections. Nonetheless, young people’s appraisal of their lives changed considerably with non-normative life events due to the pandemic in the domains of work, learning and peers.

The final hypotheses predicted that the relationship of stressors with life satisfaction varies by gender and across developmental phases in the transition to adulthood (H3). The estimates indicate overall effect heterogeneity by gender, age group, and education status, but not by living arrangement. Young women’s life satisfaction responded more strongly than young men’s to the experience of COVID-19 among family or friends and to career worries, while for males, financial strain weighed more heavily. The findings thus support previous observations that financial worries are more threatening to young men than young women (Kopasker et al., 2018). For adolescents aged 18 years or less, worries about job skills learning were stronger predictors of life satisfaction than among young adults. Life satisfaction reacted to career worries for those in education at baseline, whereas worries about job skills learning emerged as more substantial adversity among those not in education. There is thus considerable heterogeneity in how different stressors are perceived and responded to according to gender, one’s developmental stage, and economic activity. Life satisfaction among women, adolescents and those in education reacted more strongly to the stressors considered here. Maybe young people already in employment during the pandemic felt more protected, possibly due to the furlough scheme that enabled employers to retain staff between March 2020 and September 2021.

The results contribute to the literature in multiple ways. First, in line with previous research conducted in the US (Graupensperger et al., 2022), there has been a remarkable recovery in life satisfaction among young people in the UK as COVID-19 lockdown measures were lifted. Nonetheless, while the return to baseline is welcome, young people’s well-being has been on a downward trajectory for more than two decades (Gagné et al., 2021). ‘Building back better’ as outlined by the UK government (HM Treasury, 2021) will require fixing ingrained challenges that have prolonged the transition to adulthood and made career entry more uncertain and risk-laden (Gagné et al., 2022).

Career-related uncertainties, particularly for those still in education, emerged as a critical antecedent for life satisfaction. Previous research has identified disruptions to social relations as paramount for the widespread decline in well-being during the first phase of the pandemic (Graupensperger et al., 2022). In the current sample, career-related worries of disrupted prospects and lost job skills learning were at least as strong predictors of life satisfaction as the stressor related to social relations. It is conceivable that differences in the research design, timing, and measure of social contacts and well-being outcome between the current and previous studies explain the different findings. However, it could also be the case that uncertainties, particularly those associated with one’s career prospects, weighed higher on young people’s minds than the frequency of interaction with others as lockdown restrictions were phased out. The findings also suggest a potential positive role of quality career development interventions for mitigating the impact of career worries on well-being. This has long been proposed in the research literature (Redekopp and Huston, 2018) but continues to receive little explicit attention in career guidance policy or practice (Cedefop et al., 2020).

Continued efforts to address pandemic-related stressors remain relevant as a non-negligible proportion of young people continues to experience disruptions due to COVID-19. More than two years after the first national lockdown in March 2020 in the UK, nearly 20 % reported fewer social contacts and 1 in 10 felt the pandemic had substantially worsened their career prospects and job skills learning, respectively. The continued prevalence of adverse pandemic-related experiences raises concerns that the recovery has not reached all young people equally. Scarring of future life chances stems predominantly from prolonged periods of lost learning (Guvenen et al., 2017). These vulnerabilities require greater attention as countries leave the acute phase of the pandemic behind.

Advancing the stress-process model, this study reveals heterogeneous effects of stressors on life satisfaction by gender and developmental stage. Adolescents’, women’s, and students’ life satisfaction reacted more strongly to the considered stressors (Ellwardt and Präg, 2021). The findings support the assumption of stress proliferation, indicating that some young people drew on additional resources to buffer perceived stressors while others could not (Pearlin et al., 2005). The results suggest that supporting well-being and life satisfaction requires solutions that provide direct and indirect support whilst considering individual resources and vulnerabilities.

The strengths of the study are the rich list of potential event-related stressors, its longitudinal design, and the removal of time-invariant individual effects, whether observed or unobserved, through fixed effects regression models.

However, like any study, there are some limitations. A relatively small sample limits the scope for more detailed subgroup analyses. It would be worthwhile to examine adversities and trajectories of well-being by more detailed measures of chronic stress exposure and markers of adulthood, including ethnicity, living with a partner, or parenthood. While having many desirable properties, fixed-effects models might lead to attenuated coefficient estimates if covariates are measured with an error or change little over time. The short time dimension of the panel also limits us to assessing the concurrent association of stressors with well-being. It is conceivable that the influence of stressors goes beyond their contemporary association with well-being if, for example, the duration of exposure matters. Finally, while overall well-balanced, the non-random nature of the sample hampers generalisability. The fixed-effects strategy overcomes some concerns by removing time-invariant factors related to sample selection, but sampling error remains technically unknown. Despite these caveats, this study provides rigorous and novel insights into the interlinkages and dynamics of COVID-19-related stressors and young people’s mental health.

## Conclusion

There is little examination of changes in youth’s life satisfaction during the second phase of the pandemic when mass vaccine programmes were rolled out, and lockdown restrictions were lifted. This matters for the understanding of processes of recovery in subjective well-being. This study shows improvements in life satisfaction among young people since February 2021. Moreover, young people reported increased social contacts, reduced concerns about career prospects and job skill learning, but persisting worries about their financial situation and future, severe cases of COVID-19 among friends and family, and increased rates of COVID-19 infections since the lifting of lockdown restrictions. Recovering social contacts and receding career-related stressors contributed to improved life satisfaction, while direct health-related stressors did not. These findings highlight the importance of considering stressors from various life domains for a more comprehensive understanding of improvements in youth’s life satisfaction as countries learn to live with Covid. The results suggest that policies that ease behavioural restrictions, emphasise economic recovery, and minimise disruptions to learning can support the recovery of life satisfaction among young people. Since career-related worries emerge as strong predictors of life satisfaction, at least on par with social contacts, and ahead of health-related stressors or financial worries, investment in good career development activities and guidance might help protect young people against future risks.

## Supplementary Information

Supplementary Information

## References

[CR1] Banerjee A, Pasea L, Harris S, Gonzalez-Izquierdo A, Torralbo A, Shallcross L, Noursadeghi M, Pillay D, Sebire N, Holmes C, Pagel C, Wong WK, Langenberg C, Williams B, Denaxas S, Hemingway H (2020). Estimating excess 1-year mortality associated with the COVID-19 pandemic according to underlying conditions and age: a population-based cohort study. The Lancet.

[CR2] Blanchflower, D. G., & Bryson, A. (2022). *The Female Happiness Paradox*. 10.3386/W29893. 10.1007/s11135-023-01628-5 PMC9942082 36844462

[CR3] Blanchflower DG, Clark AE (2021). Children, unhappiness and family finances. Journal of Population Economics.

[CR4] Burger K, Samuel R (2017). The role of perceived stress and self-efficacy in young people’s life satisfaction: a longitudinal study. Journal of Youth and Adolescence.

[CR5] Cameron, C., & Trivedi, P. (2010). *Microeconometrics using stata* (Revised edition, Issue 4). Stata Press.

[CR6] Cedefop, European Commission, ETF, ICCDPP, ILO, OECD, & UNESCO. (2020). *Career guidance policy and practice in the pandemic: results of a joint international survey – June to August 2020*. Publications Office of the European Union. http://data.europa.eu/doi/10.2801/318103.

[CR7] Chandola, T., Kumari, M., Booker, C. L., & Benzeval, M. (2020). The mental health impact of COVID-19 and lockdown-related stressors among adults in the UK. *Psychological Medicine*, 1–10. 10.1017/S0033291720005048. 10.1017/S0033291720005048 PMC7783135 33280639

[CR8] Dias, M. C., Johnson-Watts, E., Joyce, R., Postel-Vinay, F., Spittal, P., & Xu, X. (2021). *Job opportunities during the pandemic*. 10.1920/BN.IFS.2021.BN0335.

[CR9] Diener E, Oishi S, Tay L (2018). Advances in subjective well-being research. Nature Human Behaviour.

[CR10] Durayappah A (2010). The 3P model: a general theory of subjective well-being. Journal of Happiness Studies.

[CR11] Ellwardt L, Präg P (2021). Heterogeneous mental health development during the COVID-19 pandemic in the United Kingdom. Scientific Reports.

[CR12] Evandrou M, Falkingham J, Qin M, Vlachantoni A (2021). Changing living arrangements and stress during Covid-19 lockdown: Evidence from four birth cohorts in the UK. SSM - Population Health.

[CR13] Fancourt D, Steptoe A, Bu F (2021). Trajectories of anxiety and depressive symptoms during enforced isolation due to COVID-19 in England: a longitudinal observational study. The Lancet Psychiatry.

[CR14] Fouad NA, Bynner J (2008). Work transitions. American Psychologist.

[CR15] Gagné T, Sacker A, Schoon I (2022). Transition milestones and life satisfaction at ages 25/26 among cohorts born in 1970 and 1989–90. Advances in Life Course Research.

[CR16] Gagné T, Schoon I, Sacker A (2021). Trends in young adults’ mental distress and its association with employment: Evidence from the Behavioral Risk Factor Surveillance System, 1993–2019. Preventive Medicine.

[CR17] Graupensperger, S., Calhoun, B. H., Patrick, M. E., & Lee, C. M. (2022). Longitudinal effects of COVID-19-related stressors on young adults’ mental health and wellbeing. *Applied Psychology: Health and Well-Being*. 10.1111/APHW.12344. 10.1111/aphw.12344 PMC9746887 35102692

[CR18] Green, F., Henseke, G., & Schoon, I. (2021a). *The Darkest Hour? New Evidence of the learning experiences, well-being and expectations of youth during the third national lockdown in the UK*. https://www.llakes.ac.uk/wp-content/uploads/2021/04/Yeah_First_Findings_Briefing_1.pdf.

[CR19] Green, F., Henseke, G., & Schoon, I. (2021b). *Perceived Effects of the Covid-19 Pandemic on Educational Progress and the Learning of Job Skills: New Evidence on Young Adults in the United Kingdom*. https://osf.io/a69k7/.

[CR20] Guvenen F, Karahan F, Ozkan S, Song J (2017). Heterogeneous scarring effects of full-year nonemployment. American Economic Review.

[CR21] Hale T, Angrist N, Goldszmidt R, Kira B, Petherick A, Phillips T, Webster S, Cameron-Blake E, Hallas L, Majumdar S, Tatlow H (2021). A global panel database of pandemic policies(Oxford COVID-19 Government Response Tracker). Nature Human Behaviour.

[CR22] Hall, A. (2014). The Concept of Life Satisfaction. *Encyclopedia of Quality of Life and Well-Being Research*, 3599–3601. 10.1007/978-94-007-0753-5_1649.

[CR23] Henkens, J. H. D., Kalmijn, M., & de Valk, H. A. G. (2022). Life Satisfaction Development in the Transition to Adulthood: Differences by Gender and Immigrant Background. *Journal of Youth and Adolescence*. 10.1007/s10964-021-01560-7. 10.1007/s10964-021-01560-7 PMC8828595 35024977

[CR24] HM Treasury. (2021). *Build Back Better: our plan for growth*. GOV.UK. www.gov.uk/official-documents.

[CR25] Holt-White, E., & Montacute, R. (2020). *Graduate Recruitment and Access to the Workplace*. https://dera.ioe.ac.uk/36101/1/Access-to-the-Workplace-Impact-Brief.pdf.

[CR26] Iasiello M, van Agteren J, Cochrane EM (2020). Mental health and/or mental illness: a scoping review of the evidence and implications of the dual-continua model of mental health. Evidence Base: A Journal of Evidence Reviews in Key Policy Areas.

[CR27] Jeličic H, Jeličic´• J, Phelps E, Lerner RM (2010). Why missing data matter in the longitudinal study of adolescent development: using the 4-h study to understand the uses of different missing data methods. Journal of Youth and Adolescence.

[CR60] Jones, A.M., Rice, N., d’Uva, T.B., & Balia, S. (2013). Applied Health Economics (2nd ed.). Routledge. 10.4324/9780203102411.

[CR28] Kopasker D, Montagna C, Bender KA (2018). Economic insecurity: A socioeconomic determinant of mental health. SSM - Population Health.

[CR29] Levin KA, Currie C (2014). Reliability and Validity of an Adapted Version of the Cantril Ladder for Use with Adolescent Samples. Social Indicators Research.

[CR30] Liu K, Salvanes KG, Sørensen E (2016). Good skills in bad times: Cyclical skill mismatch and the long-term effects of graduating in a recession. European Economic Review.

[CR31] Loades ME, Chatburn E, Higson-Sweeney N, Reynolds S, Shafran R, Brigden A, Linney C, McManus MN, Borwick C, Crawley E (2020). Rapid systematic review: the impact of social isolation and loneliness on the mental health of children and adolescents in the context of COVID-19. Journal of the American Academy of Child & Adolescent Psychiatry.

[CR32] Lombardo P, Jones W, Wang L, Shen X, Goldner EM (2018). The fundamental association between mental health and life satisfaction: Results from successive waves of a Canadian national survey. BMC Public Health.

[CR33] Mallapaty S (2021). Will Covid become a disease of the young. Nature.

[CR34] Manwell LA, Barbic SP, Roberts K, Durisko Z, Lee C, Ware E, McKenzie K (2015). What is mental health? Evidence towards a new definition from a mixed methods multidisciplinary international survey. BMJ Open.

[CR35] Núñez-Regueiro F, Núñez-Regueiro S (2021). Identifying Salient Stressors of Adolescence: A Systematic Review and Content Analysis. Journal of Youth and Adolescence.

[CR36] ONS. (2018, September 26). *Personal well-being user guidance*. https://www.ons.gov.uk/peoplepopulationandcommunity/wellbeing/methodologies/personalwellbeingsurveyuserguide.

[CR37] ONS. (2022, March 4). *Coronavirus and the social impacts on Great Britain - Office for National Statistics*. Coronavirus and the Social Impacts on Great Britain. https://www.ons.gov.uk/peoplepopulationandcommunity/healthandsocialcare/healthandwellbeing/bulletins/coronavirusandthesocialimpactsongreatbritain/latest#measuring-the-data.

[CR38] Patalay P, Fitzsimons E (2018). Development and predictors of mental ill-health and wellbeing from childhood to adolescence. Social Psychiatry and Psychiatric Epidemiology.

[CR39] Pearlin LI, Schieman S, Fazio EM, Meersman SC (2005). Stress, health, and the life course: Some conceptual perspectives. Journal of Health and Social Behavior.

[CR40] Petersen, K. J., Humphrey, N., & Qualter, P. (2021). Dual-Factor Mental Health from Childhood to Early Adolescence and Associated Factors: A Latent Transition Analysis. *Journal of Youth and Adolescence*, 1–16. 10.1007/S10964-021-01550-9/TABLES/4. 10.1007/s10964-021-01550-9 PMC9090675 34919196

[CR41] Pierce M, Hope H, Ford T, Hatch S, Hotopf M, John A, Kontopantelis E, Webb R, Wessely S, McManus S, Abel KM (2020). Mental health before and during the COVID-19 pandemic: a longitudinal probability sample survey of the UK population. The Lancet Psychiatry.

[CR42] Pierce M, McManus S, Hope H, Hotopf M, Ford T, Hatch SL, John A, Kontopantelis E, Webb RT, Wessely S, Abel KM (2021). Mental health responses to the COVID-19 pandemic: a latent class trajectory analysis using longitudinal UK data. The Lancet Psychiatry.

[CR43] Preetz, R., Filser, A., Brömmelhaus, A., Baalmann, T., & Feldhaus, M. (2021). Longitudinal Changes in Life Satisfaction and Mental Health in Emerging Adulthood During the COVID-19 Pandemic. Risk and Protective Factors: 10.1177/21676968211042109, *9*(5), 602–617. 10.1177/21676968211042109.

[CR44] Raybould, P. (2021, August 26). *Higher education institutions are right to innovate teaching delivery, but students’ expectations must be managed to avoid disappointment - HEPI*. HEPI Blog. https://www.hepi.ac.uk/2021/08/26/higher-education-institutions-are-right-to-innovate-teaching-delivery-but-students-expectations-must-be-managed-to-avoid-disappointment/.

[CR45] Redekopp, D. E., & Huston, M. (2018). The broader aims of career development: mental health, wellbeing and work. 10.1080/03069885.2018.1513451, *47*(2), 246–257. 10.1080/03069885.2018.1513451.

[CR46] Robinson E, Daly M (2021). Explaining the rise and fall of psychological distress during the COVID-19 crisis in the United States: Longitudinal evidence from the Understanding America Study. British Journal of Health Psychology.

[CR47] Royston P, White IR (2011). Multiple Imputation by Chained Equations (MICE): Implementation in Stata. Journal of Statistical Software.

[CR48] Santomauro DF, Mantilla Herrera AM, Shadid J, Zheng P, Ashbaugh C, Pigott DM, Abbafati C, Adolph C, Amlag JO, Aravkin AY, Bang-Jensen BL, Bertolacci GJ, Bloom SS, Castellano R, Castro E, Chakrabarti S, Chattopadhyay J, Cogen RM, Collins JK, Ferrari AJ (2021). Global prevalence and burden of depressive and anxiety disorders in 204 countries and territories in 2020 due to the COVID-19 pandemic. The Lancet.

[CR49] Schmalbach, B., Zenger, M., Tibubos, A. N., Kliem, S., Petrowski, K., & Brähler, E. (2021). *Psychometric Properties of Two Brief Versions of the Hopkins Symptom Checklist: HSCL-5 and HSCL-10*. 10.1177/1073191119860910. 10.1177/1073191119860910 31272193

[CR50] Schoon I, Bynner J (2019). Young people and the great recession: Variations in the school-to-work transition in Europe and the United States. Longitudinal and Life Course Studies.

[CR51] Schoon, I., & Henseke, G. (2022). Social inequalities in young people’s mental distress during the Covid-19 pandemic: Do psychosocial resource factors matter? *Frontiers in Public Health*, *0*. 10.3389/FPUBH.2022.820270. 10.3389/fpubh.2022.820270 PMC8964111 35359768

[CR52] Sellers R, Warne N, Pickles A, Maughan B, Thapar A, Collishaw S (2019). Cross-cohort change in adolescent outcomes for children with mental health problems. Journal of Child Psychology and Psychiatry.

[CR53] Settersten RA, Bernardi L, Härkönen J, Antonucci TC, Dykstra PA, Heckhausen J, Kuh D, Mayer KU, Moen P, Mortimer JT, Mulder CH, Smeeding TM, van der Lippe T, Hagestad GO, Kohli M, Levy R, Schoon I, Thomson E (2020). Understanding the effects of Covid-19 through a life course lens. Advances in Life Course Research.

[CR54] Sharon T (2016). Constructing Adulthood: Markers of Adulthood and Well-Being Among Emerging Adults. Emerging Adulthood.

[CR55] Strand, B. H., Dalgard, O. S., Tambs, K., & Rognerud, M. (2009). Measuring the mental health status of the Norwegian population: A comparison of the instruments SCL-25, SCL-10, SCL-5 and MHI-5 (SF-36). 10.1080/08039480310000932, *57*(2), 113–118. 10.1080/08039480310000932. 10.1080/08039480310000932 12745773

[CR56] Taquet M, Luciano S, Geddes JR, Harrison PJ (2021). Bidirectional associations between COVID-19 and psychiatric disorder: retrospective cohort studies of 62 354 COVID-19 cases in the USA. The Lancet Psychiatry.

[CR57] Thoits PA (2010). Stress and Health: Major Findings and Policy Implications. Journal of Health and Social Behavior.

[CR58] Wright, L., Steptoe, A., & Fancourt, D. (2020). How are adversities during COVID-19 affecting mental health? Differential associations for worries and experiences and implications for policy. *MedRxiv*, 2020.05.14.20101717. 10.1101/2020.05.14.20101717.

