# Supplementary material for: Living with COVID-19: Subjective Well-Being in the Second Phase of the Pandemic
Source: J Youth Adolesc. 2022 Jul 4;51(9):1679–92. doi: 10.1007/s10964-022-01648-8 (PMC9252564; doi:10.1007/s10964-022-01648-8)
Supplement: Supplementary file 1 — Supplementary Information [file 10964_2022_1648_MOESM1_ESM.docx]

# Appendix

Table A1: Descriptive Statistics (in %)

| **Variable** | **YEAH SAMPLE** | **UK-HLS COVID study** | **UK LFS (Q1/2021-Q3/2021, incl. proxy)** |
| --- | --- | --- | --- |
| **Age** |  |  |  |
| 16-18 | 22.4 | 21.5 | 28.5 |
| 19+ | 77.6 | 78.5 | 71.5 |
| **Gender** |  |  |  |
| Male | 48.7 | 30.3 | 51.1 |
| Female | 51.3 | 69.8 | 48.9 |
| **Region** |  |  |  |
| Northeast & Yorkshire | 13.3 | 12.0 | 12.9 |
| Northwest | 11.0 | 9.9 | 10.9 |
| Midlands | 17.2 | 17.7 | 16.5 |
| Southwest & Wales | 11.9 | 13.6 | 13.8 |
| Southeast & Anglia | 21.7 | 21.8 | 21.8 |
| Greater London | 14.8 | 13.1 | 12.9 |
| Scotland | 8.3 | 7.4 | 8.2 |
| Northern Ireland | 1.9 | 4.6 | 2.8 |
| **Ethnicity** |  |  |  |
| White | 75.6 | 76.5 | 82.6 |
| Black, Asian, minority ethnic | 24.4 | 23.5 | 17.4 |
| **Limiting health condition** |  |  |  |
| No | 75.0 | 75.7 | 82.2 |
| Yes | 25.0 | 24.3 | 17.8 |
| **Employment Status** |  |  |  |
| In Education | 43.5 |  | 46.0 |
| In Work | 46.1 |  | 41.6 |
| Not in Employment, Education or Training (NEET) | 10.4 |  | 12.4 |
| **Educational Attainment** |  |  |  |
| Below Level 2/Other | 7.4 |  | 13.5 |
| GCSE or eq. | 16.0 |  | 28.6 |
| A levels or eq. | 42.0 |  | 34.1 |
| Level 4 or above | 34.6 |  | 23.8 |
| **Subjective Financial Situation** |  |  |  |
| Living comfortably | 19.6 | 30.6 |  |
| Doing alright | 38.1 | 49.8 |  |
| Just about getting by | 26.5 | 14.6 |  |
| Finding it difficult | 15.8 | 5.0 |  |
| **Living with parents/ guardians** |  |  |  |
| Yes | 57.5 | 76.5 | 74.6 |
| No | 42.6 | 23.5 | 25.4 |
| **Household social grade** |  |  |  |
| Managerial & professional | 43.2 |  | 38.5 |
| Intermediate | 23.5 |  | 20.4 |
| Working-class/ Not in work | 33.3 |  | 41.1 |
| N | 6,000 | 1,441 | 28,722 |
